# Strain-Specificity and Disease-Specificity of Probiotic Efficacy: A Systematic Review and Meta-Analysis

**DOI:** 10.3389/fmed.2018.00124

**Published:** 2018-05-07

**Authors:** Lynne V. McFarland, Charlesnika T. Evans, Ellie J. C. Goldstein

**Affiliations:** ^1^Department of Medicinal Chemistry, School of Pharmacy, University of Washington Medical Center, Seattle, WA, United States; ^2^Department of Preventive Medicine and Center for Healthcare Studies, Feinberg School of Medicine, Northwestern University, Chicago, IL, United States; ^3^Department of Veterans Affairs (VA), Center of Innovation of Complex Chronic Healthcare (CINCCH), Edward Hines Jr VA Hospital, Hines, IL, United States; ^4^RM Alden Research Laboratory, David Geffen School of Medicine at UCLA, Los Angeles, CA, United States

**Keywords:** strain specificity, disease specificity, probiotic strains, meta-analysis, pooling data, antibiotic-associated diarrhea, *Clostridium difficile*, *Saccharomyces*, *Lactobacillus*

## Abstract

**Background:**

As the use and diversity of probiotic products expands, the choice of an appropriate type of probiotic is challenging for both medical care professionals and the public alike. Two vital factors in choosing the appropriate probiotic are often ignored, namely, the probiotic strain-specificity and disease-specificity for efficacy. Reviews and meta-analyses often pool together different types of probiotics, resulting in misleading conclusions of efficacy.

**Methods:**

A systematic review of the literature (1970–2017) assessing strain-specific and disease-specific probiotic efficacy was conducted. Trials were included for probiotics with an identifiable strain (either single strain or mixtures of strains) that had at least two randomized, controlled trials for each type of disease indication. The goal was to determine if probiotic strains have strain and/or disease-specific efficacy.

**Results:**

We included 228 trials and found evidence for both strain specificity and disease specificity for the efficacy of specific probiotic strains. Significant efficacy evidence was found for 7 (70%) of probiotic strain(s) among four preventive indications and 11 (65%) probiotic strain(s) among five treatment indications. Strain-specific efficacy for preventing adult antibiotic-associated diarrhea was clearly demonstrated within the *Lactobacillus* species [e.g., by the mixture of *Lactobacillus acidophilus* CL1285, *Lactobacillus casei* LBC80R, and *Lactobacillus rhamnosus* CLR2 (Bio-K+^®^), by *L. casei* DN114001 (Actimel^®^) and by *Lactobacillus reuteri* 55730], while other *Lactobacillus* strains did not show efficacy. Significant disease-specific variations in efficacy was demonstrated by *L. rhamnosus* GG and *Saccharomyces boulardii* CNCM I-745, as well as other probiotic strains.

**Conclusion:**

Strong evidence was found supporting the hypothesis that the efficacy of probiotics is both strain-specific and disease-specific. Clinical guidelines and meta-analyses need to recognize the importance of reporting outcomes by both specific strain(s) of probiotics and the type of disease. The clinical relevance of these findings indicates that health-care providers need to take these two factors into consideration when recommending the appropriate probiotic for their patient.

## Introduction

The use of probiotics has become increasingly popular across the world and probiotic use in hospitalized patients may reach as high as 55% in admitted patients (1). Probiotics are defined as “live microorganisms that, when administered in adequate amounts, confer a health benefit on the host” (2), but unfortunately, this definition does not provide any practical guidance when choosing a probiotic. Decades of clinical trials have provided a foundation for a diverse array of probiotics (either single strain or multi-strain mixtures), but matching the appropriate probiotic strain or mixture to the patient’s need has been challenging (3).

Recently, research has supported the concept that not all probiotics are equally effective, but a consensus has not been uniformly reached as to which probiotic product should be used for specific disease conditions (4, 5). Distinguishing the different probiotic products is challenging due to differences in their mechanisms-of-action, manufacturing processes, quality control of the product, and efficacy by different strain(s). Differences in strain-specific efficacy began to be reported in 2010 as genomic analysis characterized bacterial and fungal strains in greater detail (6, 7). International probiotic guidelines and recognized experts in the field started to recommend using strain designations when reporting outcomes in clinical trials so that strain-specific efficacy can be determined, but this recommendation has not been uniformly followed (2, 4, 8, 9). *In vitro* assays and animal model data indicate efficacy differs from strain to strain among tested potential probiotic strains (10). Screening tests include determining survival from ingestion to the target organ (most commonly the intestinal tract) using pharmacokinetic studies, ability to interfere with pathogenesis (typically using animal models of disease), and stability of the microbe preparation (11). Domig et al. screened over 127 different *Lactobacillus* strains and found only 3% had potential as a probiotic, based on survival to the target organ and ability to resist bile and stomach acidity (12). An in-depth investigation of over 170 species of *Lactobacillus* found significant variation in sensitivities to antibiotics and ability to act as a probiotic candidate (7).

Different probiotic strains have different mechanisms-of-action against pathogens including: bacteriocins that directly kill or inhibit specific pathogens, the destruction of pathogenic toxins, reinforcement of the integrity of host cells (such as intestinal enterocytes), interference with pathogen attachment to host cells (termed “colonization resistance” or the barrier effect), restoration of dysbiosis of the normal microflora, and the ability to upregulate or downregulate the immune response (13). Not all probiotic strains have each of these capabilities, but several probiotics possess multiple anti-pathogen properties, such as *Saccharomyces boulardii* CNCM I-745 (14). The presence or absence of the different factors by different strains of probiotics may also explain why some probiotics are effective in some types of diseases, yet, are not effective in a different type of disease.

The goal of this systematic review and meta-analyses is to explore the efficacy of probiotics by strain and disease specificity. We gathered evidence from intervention trials randomizing adult or children subjects to either a probiotic or a control for the prevention or treatment of specific diseases.

## Methods

### Search Strategy

Prior meta-analyses were used as data sources and an updated search (through February 2017) was conducted for subsequent trials (15, 16). A search of PubMed (1960–2017), EMBASE (1974–2017), Cochrane Database of Systematic Reviews (1990–2017), ISI Web of Science (2000–2017), and three on-line clinical trial registries: Cochrane Central Register of Controlled trials,^1^ MetaRegister of Controlled Trials,^2^ and National Institutes of Health^3^ was conducted. Bibliographies of all relevant studies and conference abstracts were also reviewed. Search terms included: probiotics, randomized clinical trials (RCTs), antibiotic-associated diarrhea (AAD), *Clostridium difficile* infections (CDI), irritable bowel syndrome (IBS), inflammatory bowel disease (IBD), *Helicobacter pylori*, nosocomial infections, travelers’ diarrhea, and acute pediatric diarrhea.

### Inclusion/Exclusion Criteria

Randomized, controlled trials in adults or children were included if they were of high quality, well-described, with defined outcomes. RCTs were also only included for probiotics with identifiable strain(s) and there were at least two RCTs within specific disease indications. Indications with the most robust numbers of trials were for the prevention (pediatric or adult AAD, CDI, nosocomial infections, and travelers’ diarrhea) or treatment (CDI, IBD, IBS, *H. pylori* infections, and acute pediatric diarrhea) of disease. Non-English articles were translated and included. Disease indications with sparse data for specific probiotic strain or mixtures of strains were not included in this review. Studies were included only if they were RCTs and graded “strong strength” using standard methodology to assess strength of evidence from intervention trials (17). Additional exclusion criteria included: reviews, kinetic or safety studies, non-randomized trials, case-control studies, duplicate reports, and trials with insufficient descriptions of the type of probiotic, preclinical studies, or mechanisms of action studies. All RCTs were reviewed by all three co-authors.

### Probiotic Strain Designations

As many clinical trials often only report the genus and species of probiotic used but not the specific strain, and taxonomy has shifted over time, we retrospectively linked the reported probiotic to the most current strain designation(s) using published articles on taxonomy or clinical trials, information from manufacturer’s websites, or from communication with authors or sponsoring agencies. In some cases where the original strain was not reported in the original article but the manufacturer was known, the manufacturer was contacted to confirm that the same strains were used throughout the reported clinical trials. In other cases, trials were excluded if the strain was not reported in the original paper and the specific strain could not be retrospectively traced because the manufacturer was not reported and communications with the authors was either not productive or the authors did not remember the original source of the probiotic.

### Efficacy Assessments

Efficacy was based on documenting at least two RCTs published in peer-reviewed journals that found a significant (*p* < 0.05) reduction of either the incidence of disease (prevention trials) or a reduction in clinical symptoms (treatment trials). We stratified our descriptive assessment of efficacy into two categories: (1) more net number of RCTs with significant outcomes/disease indication (*p* < 0.05) compared with non-significant findings and (2) strain(s) with at least two RCTs with significant outcomes/disease indication, regardless of the number of non-significant trials. Total net number of significant RCTs was calculated summing the RCTs with significant efficacy findings subtracting total number of RCTs with non-significant (*p* > 0.05) outcomes.

### Meta-Analyses

As the above descriptive method presents an unweighted assessment of efficacy by comparing the total number of RCTs with significant and non-significant findings, we also conducted meta-analyses when possible. This required at least two RCTs for each sub-group of the same strains of probiotic (or mixtures) within the same type of disease. Pooled relative risks (RR) and 95% confidence intervals (CI) were calculated and heterogeneity was evaluated using the *I*^2^ statistic, using standard methods (15).

## Results

**Figure 1:**
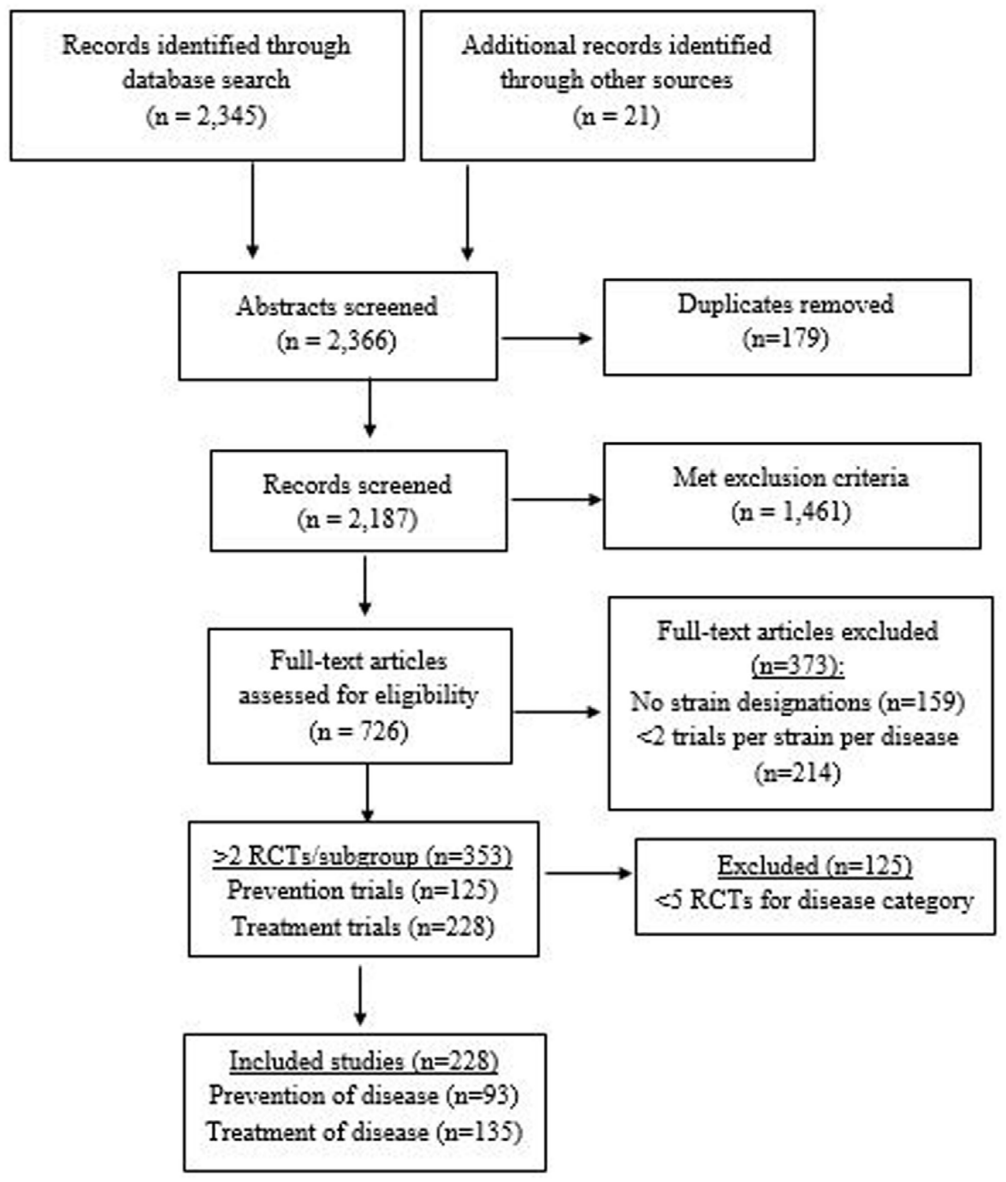
PRISMA flow diagram of evaluated studies for randomized controlled trials for probiotic strain and disease-specificity, searched from inception of databases to February 2017.

A total of 2,366 abstracts were screened (2,345 from database searches and 21 from meeting abstracts or journals not included in the above databases). A total of 726 full articles were assessed (Figure 1). Of those, 373 were excluded due to either only one RCT/strain/disease (*n* = 214) or inability to identify the strain designation (*n* = 159). A total of 353 RCTs were found with at least two RCTs per sub-group (*n* = 125 preventive trials and *n* = 228 treatment trials). Fourteen RCTs of AAD also documented CDI as a secondary outcome. Eighteen RCTs with *H. pylori*-infected patients designated eradication of *H. pylori* as their primary outcome, but also documented the prevention of adverse events and/or AAD as additional outcomes. When disease indications with the most RCTs were selected (prevention of AAD, CDI, nosocomial infections, travelers’ diarrhea, and treatment of IBD, IBS, CDI, acute pediatric diarrhea, and eradication of *H. pylori*), a total of 228 RCTs were included in this review. Probiotic trials with sparse data for each strain/disease indication subgroup were excluded (*n* = 125). A total of 25 different types of probiotics (15 single strained probiotics and 10 multi-strained mixtures) were assessed.

### Determination of Strain-Specificity

#### Strain Designations and Shifting Taxonomy

We found several challenges when identifying probiotic strain types from RCTs conducted over time: (1) shifting taxonomy of bacterial and fungal nomenclature as more precise and definitive tools (such as PCR probes and genomic sequencing) have been developed, (2) the lack of a global standard for naming strains, and (3) the incomplete identification of probiotic strains in reported RCTs publications (18). Strains are designated by a variety of naming regimes including manufacturer codes, depository numbers [such as American Type Culture Collection (ATCC) or Collection Nationale de Cultures de Microorganisms (CNCM)], or by the person isolating the original strain (for example, *Lactobacilli casei* Shirota). Genomic phenotyping has shifted microbes from one group to another or reclassified them into separate groups (such as *S. boulardii* CNCM I-745 Florastor^®^ versus *S. boulardii* Kirkman) (6) or the separation of *Lactobacillus acidophilus* into 20 separate *Lactobacillus* species groups (10). Examples of different types of probiotic products whose taxonomy has changed over the years are presented in Table 1. As the brand names of probiotic products often vary by country or by formulation, we present only the most commonly used brand names. Our initial literature search found over 700 RCTs for probiotics and various clinical indications. Typically, many published trials and meta-analyses reported the probiotic either on a genus (e.g., “a *Lactobacillus* probiotic”) or on a species level (e.g., *L. acidophilus*), but did not provide the specific strain designation (e.g., *L. acidophilus* CL1285).

**Table 1:** Changing taxonomy of probiotic strains over time.

Probiotic brand name^a^	Older designations	Current designations
Actimel^®^	*Lactobacillus casei* Immunitas *L. casei* Defensis	*L. casei* CNCM I-1518 (DN114-001)
Activia^®^	*B. lactis or B. lactis* Regularis	*B. animalis* spp*. lactis* DN173 010 (CNCM I-2494)
Bio-K + ^®^	*L. acidophilus* CL1285 and *L. casei* LBC80R	*L. acidophilus* CL1285 and *L. casei* LBC80R and *L. rhamnosus* CLR2
Culturelle^®^	*L. rhamnosus* GG	*L. rhamnosus* GG (ATCC 53103)
Dicoflor^®^	*L. rhamnosus* GR1 and *L. fermentum* RC14	*L. rhamnosus* GR1 and *L. reuteri* RC14
Florastor^®^	*Saccharomyces cerevisiae boulardii *S. boulardii* lyo *S. boulardii* 17 *S. boulardii* Hansen CBS-5926*	*S. boulardii* CNCM I-745 (ATCC 74012)
Ganeden BC	*L. sporogenes*	*Bacillus coagulans* GBI-30, 6086
Lacidofil^®^	*L. rhamnosus* R11 or LB24 and *L. acidophilus* R52 or YS or K1, or K300	*L. rhamnosus* R11 (CNCM I-1720) *L. helveticus* R52 (CNCM I-1722)
Lactinex^®^	*L. bulgaricus* and *L. acidophilus*	*L. helveticus* (ATCC 33409) and *L. gasseri* (ATCC 4962)
Probi AB^®^ or ProViva^®^	*L. plantarum*	*L. plantarum* 299v (DSM 9843)
Protecflor^®^	*B. longum* RW001 and *L. rhamnosus* R11 and *L. acidophilus* R52 and *S. boulardii*	*B. longum* R175 (CNCM I-755) and *L. rhamnosus* R11 (CNCM I-1720) and *L. helveticus* R52 (CNCM I-1722) and *S. cerevisiae boulardii* (CNCM I-1079)
Protectis^®^	*L. reuteri* DSM 55730 or *L. reuteri* SD2112 or *L. reuteri* ATCC 55730	*L. reuteri* DSM17938 or ATCC7938 (daughter strain)
Yakult^®^	*L. casei* YIT9029	*L. casei* Shirota
VSL#3^®^	*B. longum* DSM24736, *Bifidobacterium infantis* SD5220/DSM24737, *B. breve* DSM24732, *L. acidophilus* DSM24735, *L. plantarum* DSM24730, *L. paracasei* DSM24733, *L. delbrueckii* spp *bulgaricus* DSM24734, *Strept. thermophilus* DSM 24731	*B. longum* BL03, *B. infantis* spp*. lactis* BI04, *B. breve* BB02, *L. acidophilus* BA05, *L. plantarum* BP06, *L. paracasei* BP07, *L. helveticus* BD08 *Strept thermophilus* BT01
–	*Strept. faecalis*	*Enter. faecalis*
–	*L. acidophilus* La-1	*L. johnsonii* ATCC 33200
–	*B. infantis* 35624	*B. longum* spp. *longum* 35624
–	*B. lactis* Bb12 or *B. lactis* DSM15954	*B. animalis* spp. *lactis* Bb12 (CNCM 3446)

*^a^Brand names may vary by country or formulation, most common brand name given (–), no brand name*.

*ATCC, American Type Culture Collection, Manassas, VA, USA; B., Bifidobacterium; CBS, Centraal Bureau voor Schimmelcultures, Baarn, The Netherlands. CNCM, Collection Nationale de Cultures de Microorganismes (Institut Pasteur, Paris, France); DSM, Deutsche Sammlung von Mikroorganismen, Braunschweig, Germany; Entero., Enterococcus; L., Lactobacillus; nr, not reported; S., Saccharomyces; Strept., Streptococcus*.

#### *Lactobacillus* Strains

To examine if strain-specificity exists for *Lactobacillus* spp., we reviewed the literature for RCTs of various *Lactobacillus* strains. We found only one RCT that directly compared two different strains of the same species (*L. casei*). Dietrich et al. compared two similar commercial products (Actimel^®^ with *L. casei* DN-114001 and Yakult^®^ with *L. casei* Shirota) for the prevention of AAD (19). One strain (*L. casei* DN-114001) was significantly more effective in reducing AAD incidence than the other *L. casei* strain (6.7 and 33.3%, respectively, *p* < 0.05). Unfortunately, direct strain to strain comparisons in the same RCTs for the same type of disease indications are extremely rare.

**Figure 2:**
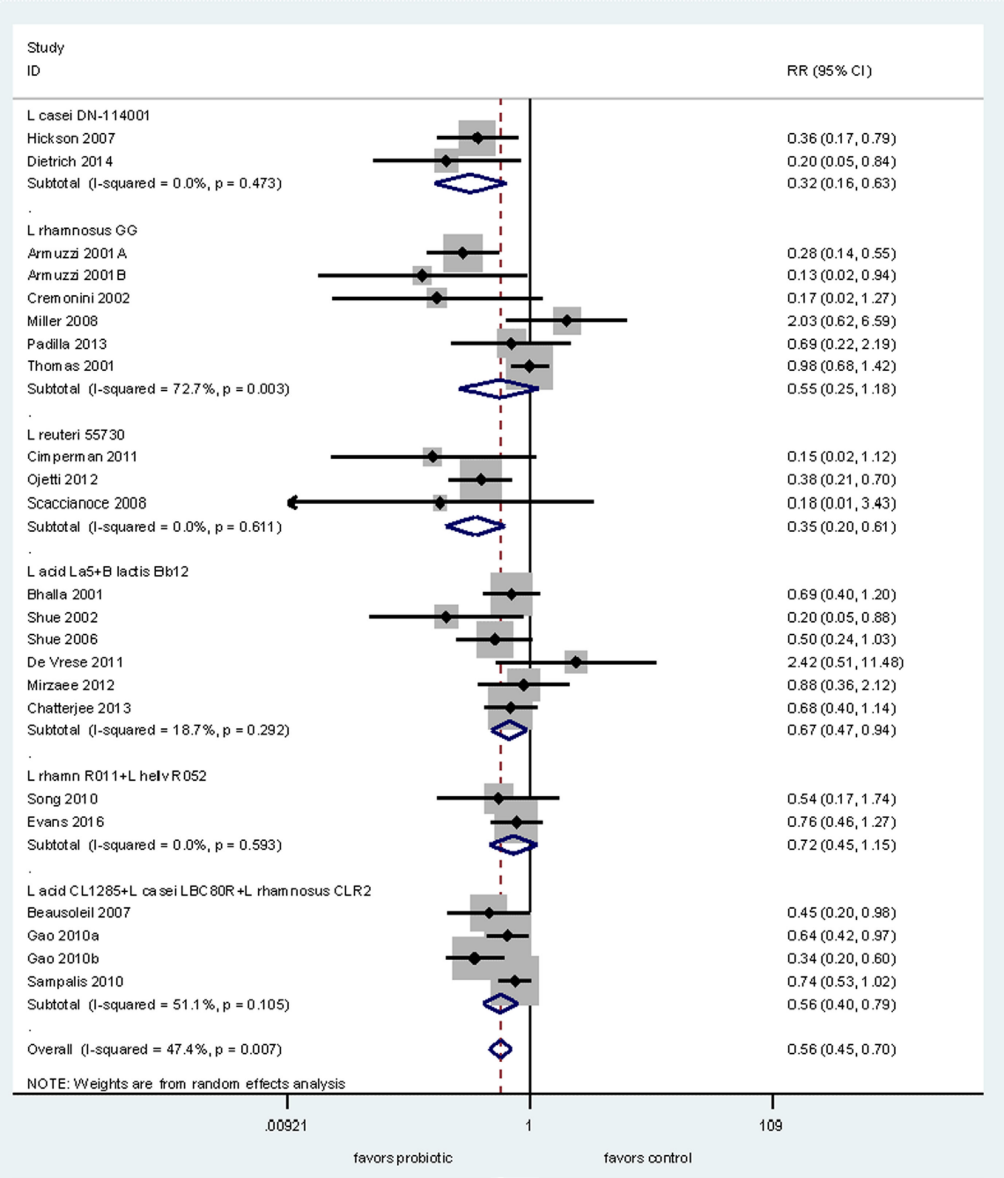
Forest plot of probiotic strain-specificity. Meta-analysis of probiotic strain-specificity for six different *Lactobacillus* probiotics for the prevention of antibiotic-associated diarrhea in adults. Meta-analysis of 22 randomized, controlled trials by sub-group of probiotic type. Abbreviations: acid, *acidophilus*; CI, confidence interval; helv, helveticus; ID, identification; L, *Lactobacillus*; rhamn, *rhamnosus*; RR, relative risk.

Next, we conducted a meta-analysis of RCTs testing probiotics within the same genus (*Lactobacillus*) for the prevention of AAD to determine if there is documented strain-specificity. We found 22 RCTs for the prevention of AAD in adults and used subgroup analyses for each of the six different *Lactobacillus* species. As shown in Figure 2, only four of six types of *Lactobacillus* strains significantly prevented AAD in adults. Pooled data from two RCTs using *L. casei* DN-114001 shows a significant reduction of AAD (RR = 0.32, 95% CI 0.16, 0.63, *I*^2^ = 0%) (19, 20). Pooled data from three RCTs using *L. reuteri* ATCC 55730 also showed a significant reduction in the incidence of AAD (RR = 0.35, 95% CI 0.20, 0.61, *I*^2^ = 0%) (21–23). In comparison, another strain (*L. rhamnosus* GG) did not show significant efficacy when data from six RCTs are pooled together (RR = 0.55, 95% CI 0.25, 1.18, *I*^2^ = 73%) (24–29). One study using *L. rhamnosus* GG reported the results of two trials, but only one had data on AAD (27). The mixture of three strains of *Lactobacillus* spp. (*L. acidophilus* CL1285, *L. casei* LBC80R, *L. rhamnosus* CLR2 or Bio-K+^®^) showed a significant reduction in AAD (RR = 0.56, 95% CI 0.40, 0.79, *I*^2^ = 51%) from data pooled from three trials with four intervention arms (30–32). Another mixture of *L. acidophilus* La5 and *B. lactis* Bb12 showed significant reduction of AAD in six RCTs in adult patients (RR = 0.67, 95% CI 0.47, 0.94, *I*^2^ = 19%) (33–38). In contrast, data from two trials using a mixture of two strains of *Lactobacillus* (*L. rhamnosus* R011 and *L. helveticus* R052) failed to find significant efficacy for the prevention of AAD (39, 40).

#### *Saccharomyces* Strains

*Saccharomyces boulardii* CNCM I-745 is a fungal single-strain probiotic with well documented efficacy for a variety of diseases (14). To demonstrate strain-specificity, we found six RCTs for two similar *Saccharomyces* species in adults with IBS (41–46). Only two RCTs with *S. boulardii* CNCM I-745 (41, 42) had comparable outcome metrics (change in symptom scores) as the two RCTs with *S. cerevisiae* I-3856 (45, 46). A significant reduction in symptom scores is seen for *S. boulardii* CNCM I-745 [weighted mean difference (WMD) = −0.72, 95% CI −1.18, −0.25, *I*^2^ = 99.2%], but not for *S. cerevisiae* CNCM I-3856 strain (WMD = −0.16, 95% CI −0.33, +0.01, *I*^2^ = 97.7%). Unfortunately, no RCTs were found that directly compared *S. boulardii* CNCM I-745 to other strains of *Saccharomyces* or for any other disease indication.

### Determination of Disease-Specificity

Another challenge is that the same probiotic strain or mixture of strains may be effective for one disease, and yet, not effective for other disease types. Indications for probiotic use are diverse, ranging from prevention of disease (for example, AAD, vaginitis, travelers’ diarrhea, sepsis, atopic dermatitis), or preventing side-effects of standard therapies for diseases (such as treatment of *H. pylori* or chemotherapy), to the treatment of acute diseases (such as *C. difficile* infections, acute pediatric or adult diarrhea, constipation) or treatment of chronic disease conditions [such as IBD, IBS, or obesity (3)].

**Figure 3:**
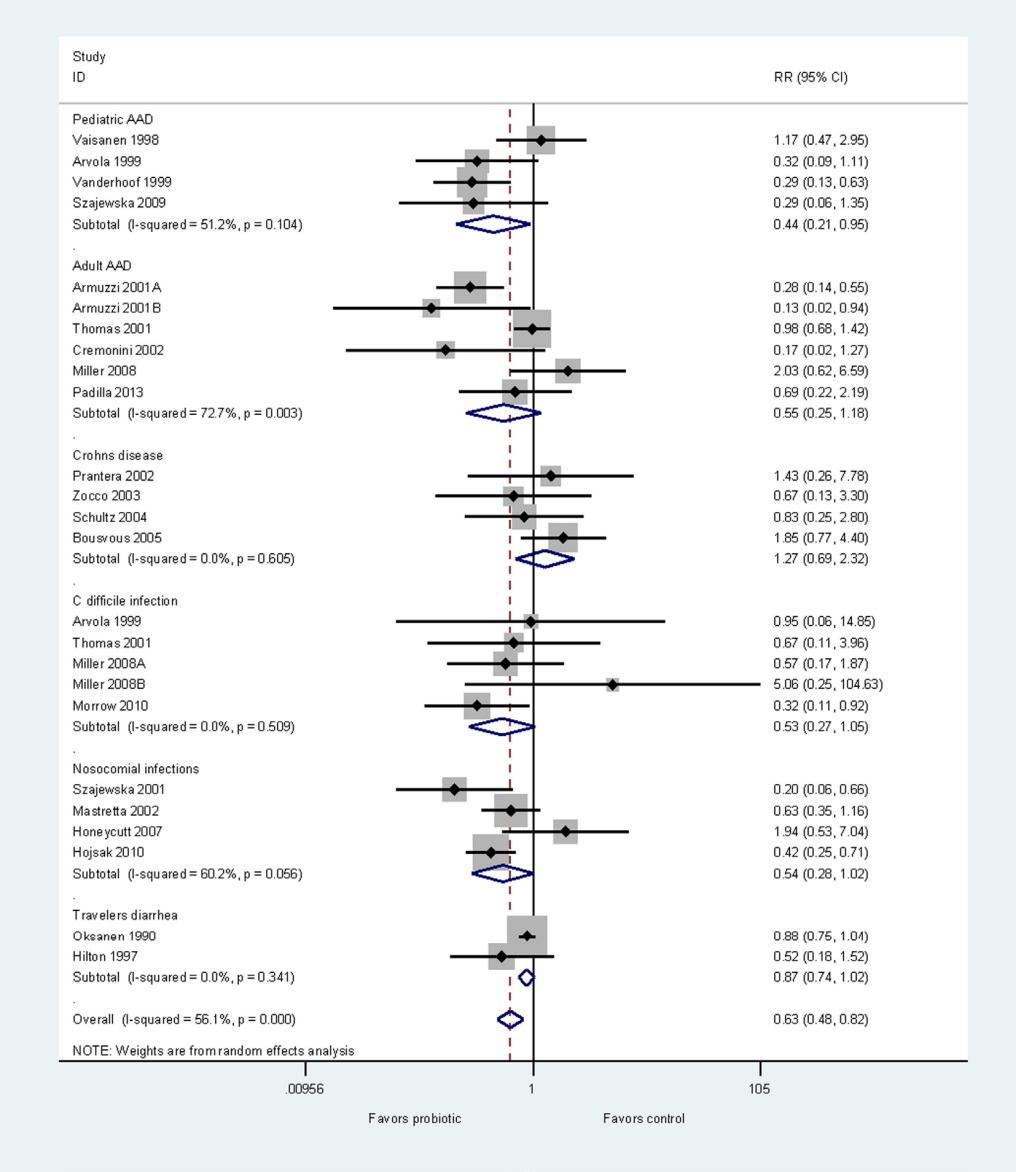
Forest plot of disease-specificity. Meta-analysis of disease-specificity shown for the prevention of six different types of diseases by one strain of probiotic, *Lactobacillus rhamnosus* GG. A meta-analysis of 24 randomized, controlled trials in adults and children. Abbreviations: AAD, antibiotic-associated diarrhea; C, *Clostridium*; ID, identification; CI, confidence interval; RR, relative risk.

To demonstrate disease specificity using one probiotic strain, we conducted a meta-analysis using only RCTs testing *L. rhamnosus* GG, and then pooled subgroups by disease type. As probiotic efficacy and disease characteristics may differ for adults and children, we assessed AAD in these two populations separately (47). Efficacy from 23 RCTs (24 treatment arms) were grouped by the type of disease indication into six groups: prevention of pediatric AAD (48–51), prevention of adult AAD (24–29), Crohn’s disease (52–55), *C. difficile* infection (27, 29, 49, 56), other types of nosocomial infections (57–60), and travelers’ diarrhea (61, 62). Three trials documented both the prevention of AAD and prevention of CDI (27, 29, 49). As shown in Figure 3, the *L. rhamnosus* GG strain has significant efficacy for the prevention of pediatric AAD (RR = 0.44, 95% CI 0.21, 0.95, *p* < 0.05). However, *L. rhamnosus* GG is not effective for other five diseases. This meta-analysis clearly demonstrates the disease-specificity of one strain of probiotic.

### Practical Application of Strain and Disease Specificity

**Figure 4:**
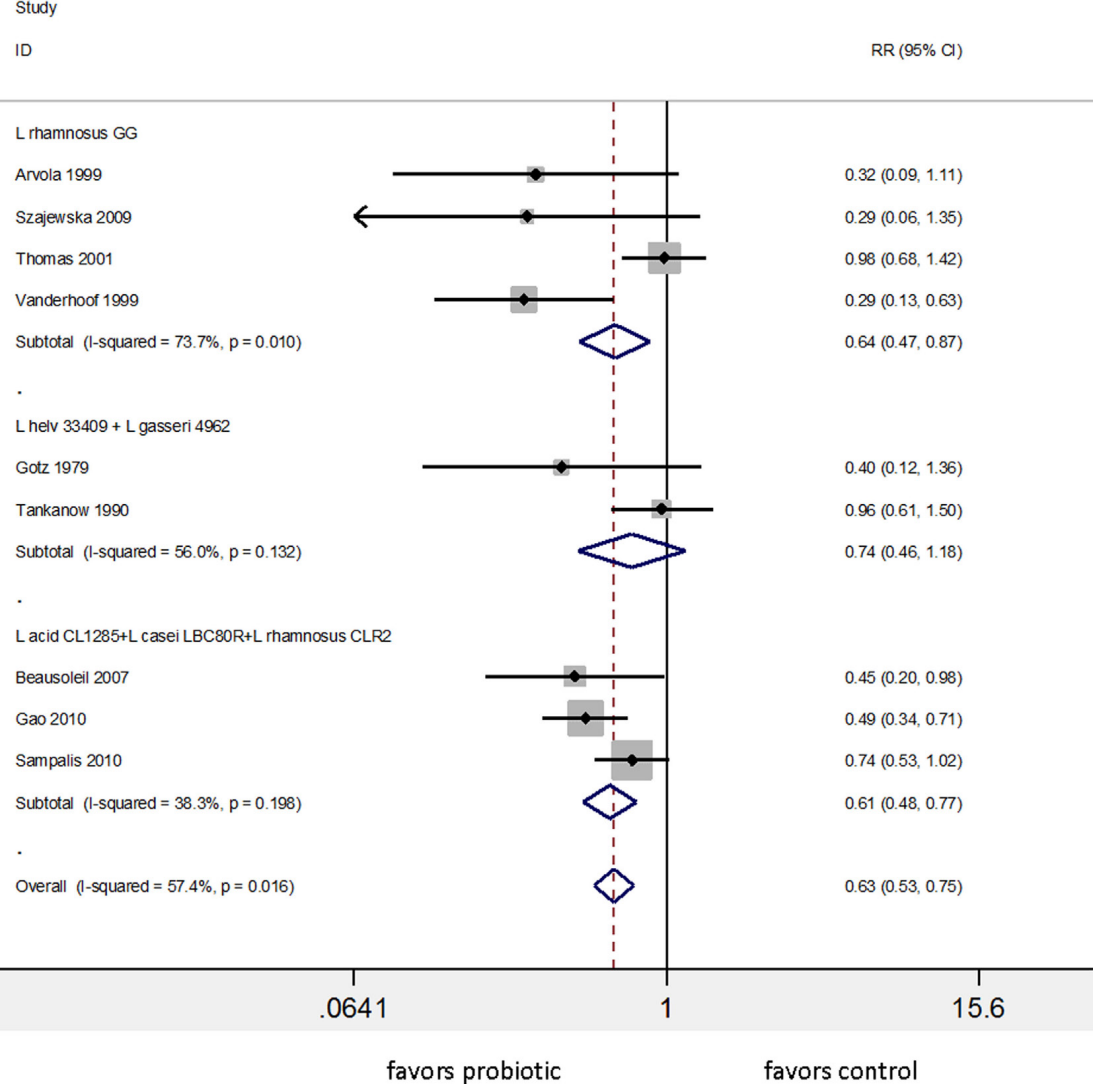
Meta-analysis of nine randomized controlled trials of three different lactobacilli-containing probiotics for the prevention of antibiotic-associated diarrhea in children and adults, sub-grouped by identical strains of *Lactobacillus*. Modified from the one “lactobacilli” group presented in Hempel et al. (64). Abbreviations: acid, *acidophilus*; CI, confidence interval; L, *Lactobacillus*; helv, *helveticus*; ID, identification; RR, relative risk.

Appreciating the importance of strain specificity and disease specificity, we reassessed reviews and meta-analyses to determine if these two factors were accounted for in their analysis and conclusions. Many meta-analyses did not account for strain-specificity correctly, as different strains were pooled within the same subgroup (63–68) and some grouped probiotics only at a genus-level (69). One meta-analysis for the prevention of AAD concluded that “the pooled RR from 62 RCTs indicated a statistically significant association of probiotic administration with reduction in AAD” (64). This conclusion was based on a pooled RR encompassing 32 different types of probiotics, and the pooled studies were a mix of treatment and prevention study designs. Hempel et al. did attempt to pool what they considered similar probiotic types into several subgroups by genus and species. Two strains were appropriately incorporated into separate sub-groups (*S. boulardii* CNCM I-745 and *Enterococcus faecium* SF68), while other sub-groups were inappropriately pooled. The subgroup called “Blend” pooled 25 RCTs of 19 different types of multi-strain mixtures and the subgroup “*Lactobacillus*” pooled 17 RCTs of 11 different species of *Lactobacillus*. Hempel et al. reported the “Lactobacilli probiotics had significant reduction in the risk of AAD (RR = 0.64, 95% CI 0.47, 0.86, *I*^2^ = 74%)” (64). As shown in Figure 4, a re-analysis of the data using sub-groups of identical strains or mixtures of strains with ≥2 RCTs showed only three subgroups of identical strain types and only two significantly prevent AAD: *L. rhamnosus* GG (RR = 0.64, 95% CI 0.47, 0.87, *I*^2^ = 74%) and one of the mixtures (*L. acidophilus* CL1285, *L. casei* LBC80R, and *L. rhamnosus* CLR2 or “Bio-K+^®^”) with RR = 0.61, 95% CI 0.48–0.77, *I*^2^ = 38%. The other mixture of *Lactobacillus* spp. (two RCTs of *L. helveticus* and *L. gasseri* mix or “Lactinex^®^”) did not significantly prevent AAD and all other lactobacilli trials reported in Hempel et al. did not have a second confirmatory trial. The importance of analyzing probiotics by species subgroups is confirmed in another meta-analysis of 22 RCTs analyzing 16 different types of probiotics for the prevention of AAD in children (70). The overall pooled data from the 22 trials appeared to confirm that probiotics were effective, but when the appropriate probiotic sub-groups were used, only two types of probiotics (*S. boulardii* CNCM I-745 pooled from five trials) and *L. rhamnosus* GG (pooled from four trials) were effective.

**Figure 5:**
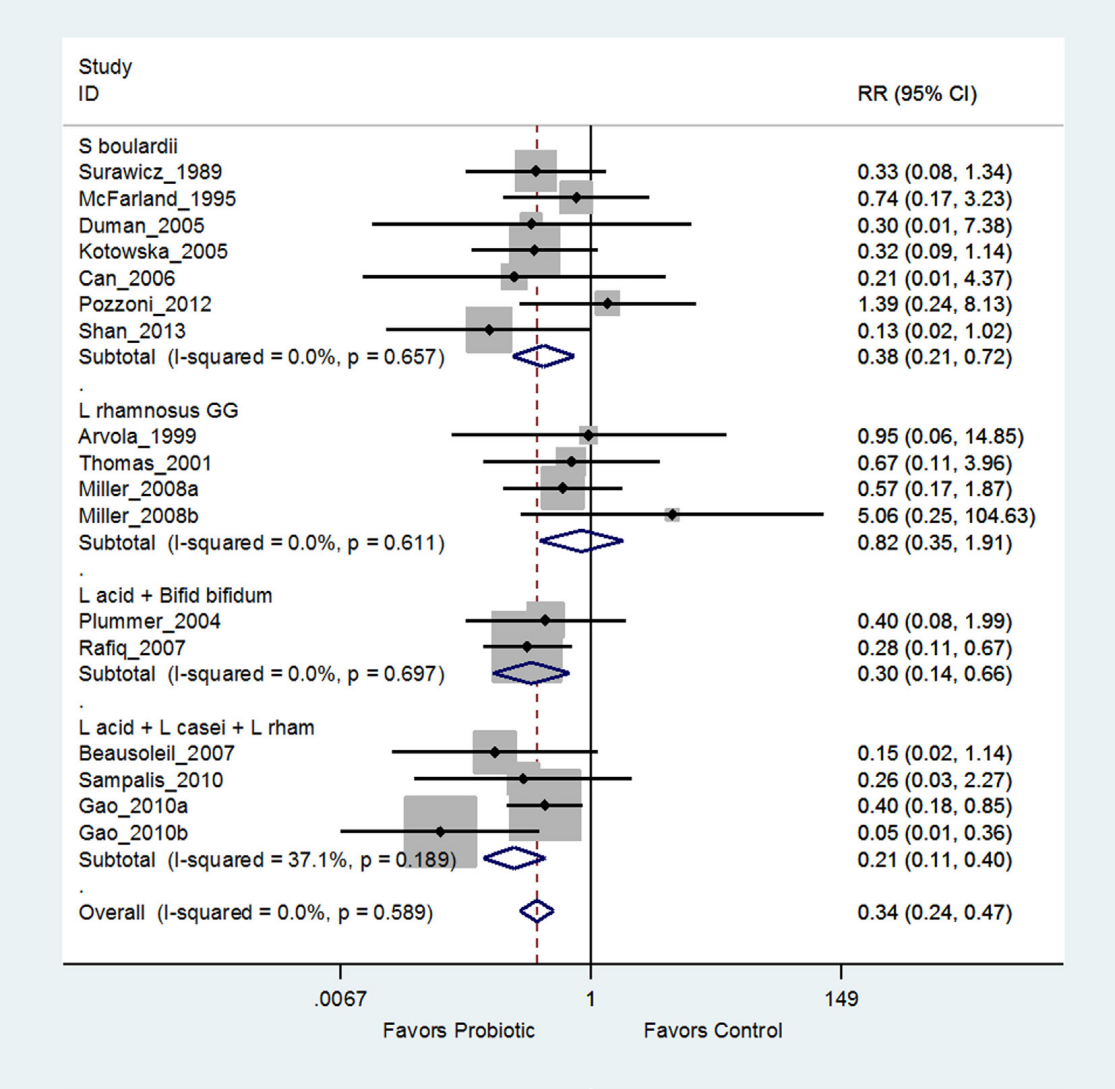
Example of forest plot from an appropriate meta-analysis using probiotic strain subgroup analysis for the prevention of *Clostridium difficile* infections modified from Lau and Chamberlain (66). Grouping by the same probiotic strain determined that *Lactobacillus rhamnosus* GG was ineffective, *S. boulardii* was effective, and only two mixtures were significantly effective (72). Abbreviations: ID, identification; CI, confidence interval; RR, relative risk; *S. boulardii, Saccharomyces boulardii* CNCM I-745; *L*., *Lactobacillus*; L acid + Bifid bifidum, *Lactobacillus acidophilus* and *Bifidobacterium bifidum* strains not reported; L acid + *L. casei* + *L. rhamn, L. acidophilus* CL1285 + *L. casei* LBC80R + *L. rhamnosus* CLR2, Bio-K+^®^.

Another recent meta-analysis of 26 RCTs concluded “Lactobacilli, mixtures, and *S. boulardii* are effective in preventing *C. difficile* infections” (66). Closer examination of the “Lactobacilli” subgroup reveals six different *Lactobacillus* strains and the “mixture” subgroup has five different mixtures of strains. Only one subgroup appropriately pooled data from seven trials using the same strain of probiotic (*S. boulardii* I-745). As shown in Figure 5, when the “Lactobacilli” and “mixture” groups are appropriately analyzed using the same strain within each sub-group, only two mixtures [(*L. acidophilus* CL1285, *L. casei* LBC80R, *L. rhamnosus* CLR2, or Bio-K+^®^) and a mix of *L. acidophilus* and *Bifidobacterium bifidum* (strains not reported)] are significantly effective for preventing *C. difficile* infections, while the other 10 types quoted as effective in the Lau and Chamberlain paper are not significantly effective or lacked a second, confirmatory trial (71).

A recent meta-analysis of 11 RCTs studying various probiotics for the control of diabetes concluded that “probiotics may be used as an important dietary supplement in reducing the glucose metabolic factors associated with diabetes” (65). The application of this conclusion would have a dramatic impact on health-care systems if probiotics were given to every patient with diabetes. Upon further assessment of the 11 trials, pooling was inappropriate for the mixtures, as there were seven different types of mixtures of strains, but pooling was appropriate for four trials, as there were two trials for *L. plantarum* DSM21380. However, when the appropriate pooling is subsequently done (72), the analysis shows no strain has a significant effect on diabetes parameters, which was contrary to the original meta-analysis conclusion.

### Prevention of Disease

The variety of different outcome metrics limited the ability to conduct a global meta-analysis for the prevention of all different diseases; therefore, we present a descriptive overview of different probiotic strains for the prevention of various diseases. We found sufficient numbers of RCTs for 10 different types of probiotics for the prevention of four common types of diseases (adult or pediatric AAD, CDI, nosocomial infections, and travelers’ diarrhea). Of the 10 types of probiotics with ≥2 RCTs/indication, 93 RCTs were assessed. Seven (70%) of the 10 probiotic types had strong strength of efficacy, as shown in Table 2. However, not every probiotic was as equally effective for every disease indication and not all strains of probiotics were tested in every type of disease. *S. boulardii* I-745 has strong evidence for the prevention of adult and pediatric AAD and travelers’ diarrhea, but not for the other types of diseases. The mixture of *L. acidophilus* CL1285, *L. casei* LBC80R, and *L. rhamnosus* CLR2 (Bio-K+^®^) also demonstrated significant efficacy for both the prevention of adult AAD and the primary prevention of CDI. Although *L. rhamnosus* GG had at least two RCTs with significant findings for the prevention of AAD and nosocomial infections, we also found an equal or more number of trials with non-significant findings. When data from all the trials are pooled for *L. rhamnosus* GG (Figure 3), significant efficacy is only found for pediatric AAD and not for the other indications. Several other single strain probiotics only had a limited number of RCTs (*n* = 2–3) for the prevention of adult AAD, but had at least two RCTs with significant findings (*L. casei* DN114001, *L. reuteri* 55730, *Enterococcus faecalis* SF68). The two-strain mixture of *L. helveticus* R52 and *L. rhamnosus* R11, Lacidofil^®^ had two RCTs with significant findings for the prevention of pediatric AAD, and had one RCT with non-significant findings (Table 2). Three other types of probiotics with ≥2 RCTs/disease had more trials with non-significant efficacy findings than trials with significant findings including: *C. butryicum* 588, *L. reuteri* 17938, and a mix of *L. acidophilus* La5 and *B. lactis* Bb12.

**Table 2:** Strength of efficacy for probiotics with identified strain designations and at least two randomized, controlled trials with significant findings for the prevention or treatment of disease.

Disease indication	Net ≥ 2 significant randomized clinical trials (RCTs) (number of significant RCTs/non-significant RCTs)	At least two RCTs with significant efficacy (number of significant RCTs/non-significant RCTs)
**Prevention**		
Adult AAD	*Saccharomyces boulardii* I-745 (11+/6−)	*Enterococcus faecalis* SF68 (2+/1−)
	*Lactobacillus acidophilus* CL1285+ *Lactobacillus casei* LBC80R+ *Lactobacillus rhamnosus* CLR2 (4+/0)^a^	*L. rhamnosus* GG (2+/4−)
	*L. casei* DN114001 (2+/0−)	*Lactobacillus reuteri* 55730 (2+/1−)

Pediatric AAD	*S. boulardii* I-745 (7+/3−)	*L. helveticus* R52 + *L. rhamnosus* R11 (2+/1−)

CDI-primary	None	*L. acidophilus* CL1285 + *L. casei* LBC80R + *L. rhamnosus* CLR2 (2+/2−)^a^

Nosocomial infections	None	*L. rhamnosus* GG (2+/2−)

Travelers’ diarrhea	*S. boulardii* I-745 (2+/0−)	

**Treatment**		
Pediatric acute diarrhea	*S. boulardii* I-745 (25+/4−)	*L. helveticus* R52 + *L. rhamnosus* R11 (2+/1−)
	*L. rhamnosus* GG (12+/3−)	
	*L. reuteri* DSN 17938 (3+/0−)	
	*L. acidophilus* LB (3+/1−)	
	*L. casei* DN114001 (3+/0−)	
	VSL#3^b^ (2+/0−)	
	*Bac. clausii* OC/SN/R (3+/1−)	

Irritable bowel syndrome	*B. infantis* 35624 (2+/0−)	*L. rhamnosus* GG (2+/2−)
	*L. plantarum* 299v (4+/1−)	*S. boulardii* I I-745 (2+/2−)
	*L. rhamnosus* GG+ *L. rhamnosus*	VSL#3^b^ (2+/2−)
	LC705 + *B. breve* Bb99 + *Prop. freudenreichii shermanii* Jc (2+/0−)	

*Helicobacter pylori* eradication	*L. helveticus* R52 + *L. rhamnosus* R11 (4+/1−)	*S. boulardii* I-745 (5+/11−)
		*L. reuteri* 55730 (2+/2−)
		*L. acidophilus* La5 + *B. animalis* spp*. lactis* Bb12 (3+/2−)

Inflammatory bowel disease	VSL#3^b^ (8+/2−)	*S. boulardii* I-745 (2+/1−)

CDI-recurrences	*S. boulardii* I-745 (2+/0−)

*AAD, antibiotic-associated diarrhea; B., Bifidobacterium; Bac, Bacillus; CDI, clostridium difficile infections; E., Enterococcus; L., Lactobacillus; Prop., Propionibacterium; S., saccharomyces*.

*^a^Includes two dose treatment arms from one trial*.

*^b^VSL#3, a mix of eight strains (*B. breve, B. longum, B. infantis, L. acidophilus, L. plantarum, L. paracasei, L. debrueckii* spp. *bulgaricus*, and *Streptococcs thermophilus*)*.

### Treatment of Disease

A descriptive overview of the efficacy of probiotic strains is also required for the treatment of various diseases, as different outcomes were used by many trials. A total of 135 RCTs for the treatment of five common types of diseases (acute pediatric diarrhea, IBS, eradication of *H. pylori*, IBD, and CDI) were found. Of the 17 types of probiotics tested, 11 (65%) had significant efficacy evidence for the treatment of these diseases. The treatment of acute pediatric diarrhea was the focus for 63 randomized trials. Seven different probiotic strains or mixtures have strong evidence for this indication (Table 2). *S. boulardii* CMCN I-745 had the most trials with significant efficacy for the treatment of acute pediatric diarrhea. Of 21 RCTs for IBS, three different probiotics had more significant trials compared to non-significant trials for the IBS (*B. infantis* 35624, *L. plantarum* 299v, and a 4-strain mixture), while three other types of probiotics had an equal number of significant and non-significant trial outcomes (Table 2). Thirty RCTs had at least two trials for each of four different types of probiotics in patients with *H. pylori* infections. One mixture (*L. helveticus* R52 + *L. rhamnosus* R11) had strong evidence for the eradication of *H. pylori* (Table 2), while three other types of probiotics (*S. boulardii* I-745, *L. reuteri* 55730, and a mixture of *L. acidophilus* La5 + *B. animalis* spp. *lactis* Bb12) had at least two RCTs showing a significant reduction in *H. pylori*. Of 13 RCTs for the treatment of IBD, two types of probiotics (VSL#3 and *S. boulardii* I-745) found significant improvement in IBD symptoms (Table 2). The strongest evidence was found for the VSL#3 mixture (*B. breve* BB02, *B. longum* BL03, *B. infantis* BI04, *L. acidophilus* BA05, *L. plantarum* BP06, *L. paracasei* BP07, *L. helveticus* BD08, *Strept. thermophiles* BT01), with a net of six trials with significant efficacy for the treatment of IBD. Fewer trials were found treating CDI infections that used prevention of recurrences as an outcome, but *S. boulardii* CNCM I-745 had a net of two significant trials.

## Discussion

Identifying an appropriate probiotic product from the diverse milieu of clinical trial evidence is a daunting challenge. The finding that efficacy is both strain-specific and disease-specific has not been clearly recognized nor acknowledged. Current literature guidelines and expert consensus now recommend reviews and meta-analyses present outcome data in appropriate probiotic strain-specific sub-groups when assessing efficacy outcomes (73, 74), but this advice is not uniformly acted upon.

This review is the first paper that we are aware of that determined strain specificity by directly comparing different probiotic strains within the same genus for one disease indication at a time and also examined the differences in efficacy of one probiotic strain for several types of diseases to determine disease-specificity. The findings from this paper support the concept that there is specific probiotic strain and disease-specific clinical efficacy. However, direct comparisons of different strains are rare and multiple trials for the same strain or mixtures for the same disease are uncommon. Despite international recommendations, published meta-analyses and reviews often fail to report strain designations and many inappropriately pool together different strains or species of probiotics into the same subgroup (64–66), or pool probiotics into the same genus level (69) when they conducted their efficacy analysis. When appropriate strain subgroups are created and the data re-analyzed, the findings often find not all the probiotic strains were effective as originally reported (65, 66, 71, 72).

One strategy to account for strain-specificity is to limit the inclusion into meta-analyses to probiotics of the same strain. Several meta-analyses have done this, either by including only trials using *S. boulardii* CNCM I-745 (75), or *L. acidophilus* LB (76), or *L. reuteri* DSM17938 (77). Szajewska et al. included trials using *L. acidophilus* LB and found a significant mean reduction in diarrhea pooled from four trials was 21.6 h (76). Urbancsek et al. limited their meta-analysis to three trials using *L. reuteri* DSM17938 and also found a significant mean reduction of diarrhea by 24.8 h (77). Yuan et al. restricted their meta-analysis to trials testing *Bifidobacterium infantis* 35624 (78), but this study was criticized for including two trials that actually used different strains of *B. infantis* SD5220 and *B. infantis* 02 (79).

Another strategy is to appropriately conduct subgroup analyses with the same probiotic strains within each sub-group (15, 16). One meta-analysis included 25 RCTs using six different types of single strain probiotics and found only one sub-group (*S. boulardii* CNCM I-745) significantly improved *H. pylori* eradication rates (RR = 1.11, 95% CI 1.07–1.16), while five other strain sub-groups (*Clostridium butyricum* 588, *L. rhamnosus* GG, *L. acidophilus* Lb, *L. reuteri* ATCC 55730, and *L. casei* DG) had no significant effect (15). Another meta-analysis of only multi-strain mixtures included 19 RCTs with six sub-groups of the same multi-strain mixes (16). Four mixtures significantly increased *H. pylori* eradication rates (*L. acidophilus* La5 and *B. animalis* spp. *lactis* Bb12; a mix of *L. helveticus* R52 and *L. rhamnosus* R11; a mix of *L. acidophilus, B. longum* and *E. faecalis* (strains not reported); and an eight-strain mixture). In our review, we found one mixture (*L. helveticus* R52 + *L. rhamnosus* R11) had four RCTs with significant reduction in *H. pylori* and only one with non-significant *H. pylori* eradication. Three other types of probiotics had RCTs showing significant reduction or non-significant eradication of *H. pylori*. However, this may not seem as dire as it sounds for the use of probiotics in *H. pylori* infected patients. A major reason for standard (non-probiotic) treatment failure in these patients is the common occurrence of side effects (diarrhea, nausea, vomiting) associated with the standard antibiotic combination treatments for *H. pylori*, resulting in high rates of non-compliance and failure to complete the entire 10–14 days of therapy. The most valuable use of specific probiotics in these patients is not the direct eradication of *H. pylori*, rather, it is for the reduction of these side-effects (as in prevention of AAD), which allows patients to complete the full course of therapy. Meta-analyses that have assessed the prevention of these side-effects also show probiotic strain specificity indicating some strains are effective for these outcomes, while others are not (15, 16).

The clinical application of the appropriate probiotic type for patients is challenging. Our review demonstrates the importance of considering both the probiotic strain specificity and how the probiotic will be applied (disease specificity). A specific probiotic type or formulation that is effective for one disease indication may not be effective for another. Some probiotic strains were found to be effective for preventing disease, but not as a treatment for disease. It was interesting to find probiotic strain-specificity within diseases that may share a similar mechanism of action for probiotic action (such as the modulation of inflammatory response in IBD or IBS). Different probiotic types were more effective for each of the inflammatory disease conditions. Strain and disease specificity may also be dependent on how the probiotic can exert health benefits (for example, differences in mechanisms-of-action) or on the ability to restore the host’s normally protective microbiota. This area of research requires further delineation.

Limitations for assessing strain-specificity and disease-specificity included the limited number of trials using the same strain or mixtures for each specific disease condition, the changing taxonomy of bacterial and fungal species for strain designations, and the lack of a global consensus on strain designations. Strain designations are often not reported in the original publications and involved tracing the history of the probiotic product development to determine strain designations. Meta-analyses and reviews need to pool strains and diseases together only when appropriate. Another limitation was the diversity of outcome metrics used within the same strain for specific diseases, thus limiting the ability to pool outcomes from multiple studies. This is the value of the descriptive overview comparing the number of studies with significant versus non-significant outcomes. Another limitation in this review is that some disease indications are diverse (such as IBD) and some probiotics may be more effective for some subgroups of disease (for example, ulcerative colitis versus Crohn’s disease versus pouchitis), but the analysis was limited by the low numbers of trials within those categories.

## Conclusion

Evidence from this review shows that there is clear strain-specificity and disease-specificity for probiotic products and every effort should be made to report specific probiotic strains or mixture of strains when analyzing the efficacy and safety of probiotics. The clinical choice of the appropriate probiotic for each patient is challenging and requires both consideration of the type of probiotic strain(s) given and the type of disease indication for which it is needed. However, there is strong evidence for the efficacy of specific probiotics for several diseases (AAD, CDI, IBD, IBS, TD, acute pediatric diarrhea, and for *H. pylori* infections).

## Author Contributions

Conceived and designed the review and meta-analysis: LM. Reviewed included papers, wrote and reviewed the paper: LM, CE, and EG.

## Conflict of Interest Statement

LM, CE, and EG serves or has served on the Bio-K Plus advisory board. EG is on the infection control and medical advisory board for Kindred Healthcare System. LM is or has been a paid lecturer for Biocodex and Lallemand and is a board member of the Biocodex Microbiome Foundation.

## Footnotes

^1^http://www.cochrane.org (Accessed: February 14, 2017).

^2^www.isrctn.com/page/mrct (Accessed: February 14, 2017).

^3^http://www.clinicaltrials.gov (Accessed: February 14, 2017).
